# Statistical Inference for the Entropy of the Transmuted Weibull Distribution Under Progressive Type-II Censored Samples

**DOI:** 10.3390/e28070794

**Published:** 2026-07-13

**Authors:** Yanqiu Zeng, Xinyu Wu, Shixiao Xiao

**Affiliations:** Chengyi College, Jimei University, Xiamen 361021, China; yqzeng1982@jmu.edu.cn (Y.Z.);

**Keywords:** Transmuted Weibull Distribution, Shannon entropy, progressive Type-II censoring, maximum likelihood estimation, Bayesian estimation, MCMC, highest posterior density interval

## Abstract

This paper investigates statistical inference for the Shannon entropy of the Transmuted Weibull Distribution under progressively Type-II censored samples. The Transmuted Weibull Distribution is obtained by applying the quadratic rank transmutation map to the cumulative distribution function of the two-parameter Weibull distribution, thereby substantially enhancing its modeling flexibility while preserving the analytical tractability of the baseline distribution. Consequently, it provides greater flexibility for modeling lifetime data exhibiting pronounced skewness and complex hazard rate behaviors. First, a closed-form expression for the Shannon entropy of the Transmuted Weibull Distribution is derived. From a frequentist perspective, the maximum likelihood estimators of the model parameters are obtained numerically using the Newton–Raphson algorithm, and the corresponding maximum likelihood estimator of Shannon entropy is derived through the invariance property of maximum likelihood estimation. To quantify estimation uncertainty, asymptotic confidence intervals are constructed using the Delta method together with the observed Fisher information matrix, while Bootstrap confidence intervals are also developed to improve finite-sample inference. From a Bayesian perspective, posterior inference is conducted using a hybrid Gibbs sampling algorithm within the Markov chain Monte Carlo framework. Bayesian point estimators of Shannon entropy are obtained under the squared error loss function, the absolute error loss function, and the 0–1 loss function, corresponding to the posterior mean, posterior median, and posterior mode, respectively. In addition, highest posterior density credible intervals are constructed for the Shannon entropy. The proposed methods are evaluated through an extensive Monte Carlo simulation study under three representative progressively Type-II censoring schemes. Estimation performance is assessed in terms of bias, mean squared error, interval coverage probability, and average interval length. The simulation results demonstrate that the Bayesian estimators consistently outperform the maximum likelihood estimator, particularly for small sample sizes and heavy censoring, while the highest posterior density credible intervals achieve more accurate coverage probabilities and shorter interval lengths. Finally, the proposed inferential procedures are illustrated using a real dataset consisting of remission times from 128 bladder cancer patients, demonstrating their practical applicability and robustness.

## 1. Introduction

In the field of reliability engineering and survival analysis, the Weibull distribution has been widely adopted due to the flexibility of its shape parameter. However, when dealing with complex data such as non-monotonic failure rates or highly skewed distributions, the traditional Weibull distribution may provide inadequate fit [1,2]. Aryal and Tsokos et al. [3] were the first to propose the Transmuted Weibull Distribution (TWD), embedding the conventional two-parameter Weibull model within a broader parametric family. By applying a quadratic transformation to the cumulative distribution function, the TWD enhances modeling flexibility while preserving the tractability of the baseline distribution. The introduction of the TWD has generated considerable research interest. Khan and King et al. [4] extended the transmutation technique to the Transmuted Weibull distribution, giving rise to the Transmuted Modified Weibull Distribution. Merovci et al. [5] investigated the properties of the transmuted Rayleigh distribution. Pobočíková et al. [6] conducted a systematic study of the statistical properties of the TWD and validated its effectiveness in reliability modeling through real-world data. Yousaf et al. [7] examined parameter estimation for the TWD under various loss functions from a Bayesian perspective. Ahmad and Ahmad [8] explored in depth the structural properties of the TWD.

In many experimental studies, particularly when analyzing the occurrence of specific events such as animal death or equipment failure, censoring is often unavoidable [9]. In lifetime analysis and reliability research, Progressive Type-II Censoring (PC-II) is a common and flexible data collection scheme, especially well-suited for practical applications where resources are limited or testing periods are lengthy [10]. Compared with conventional Type-I and Type-II censoring, PC-II censoring allows surviving units to be dynamically removed during the experiment, thereby achieving a better balance between experimental cost and inferential precision [11]. Suppose the initial sample size is n, a lifetime test is conducted on these units, and m failure observations are recorded. During the experiment, when the i failure observation $X_{i:m:n}$ is recorded as $X_{i:m:n}$, $R_{i}$ surviving units are simultaneously removed from the remaining unfailed units, where i=1,2,…,m. The experiment continues until the m failure observation is recorded, at which point the experiment terminates. Accordingly, $X_{1:m:n},X_{2:m:n},\ldots ,X_{m:m:n}$ are referred to as a Progressive Type-II censored sample, abbreviated as a PC-II sample; $X_{1:m:n},X_{2:m:n},\ldots ,X_{m:m:n}$ are the corresponding observed values. $R_{1},R_{2},\ldots ,R_{m}$ are referred to as the Progressive Type-II censoring scheme. Elsherpieny et al. [12] investigated, under Progressive Type-II censored samples, the statistical mechanism of optimizing experimental resources by removing surviving individuals at pre-specified failure points. Given the application of this scheme in lifetime prediction of electronic components and new materials [13], researchers have further pursued statistical inference for TWD parameters under Progressive Type-II censored samples.

Under the TWD, due to the presence of complex logarithmic and exponential terms in the density function, closed-form solutions for parameter estimation and entropy computation are often unattainable. This phenomenon is widespread among heavy-tailed or shape-varying distributions, and consequently poses significant challenges for statistical inference. To address this, researchers have developed a variety of methodological approaches tailored to different distributional settings. Shannon entropy is a central concept in information theory, serving as a measure of the uncertainty inherent in the information contained in a random variable. Since its introduction by Shannon in 1948, the concept has permeated numerous fields including statistical inference, signal processing, and survival analysis. Kayal and Kumar [14] were the first to systematically investigate the estimation of Shannon entropy for several shifted exponential distributions sharing a common scale parameter; they established the inadmissibility of the optimal scale-equivariant estimator under the squared error loss function and proposed an improved Stein-type estimator. Kayal et al. [15] further extended this problem to the linear exponential loss function and studied the estimation of Rényi entropy. Hassan et al. [16] examined the estimation of entropy for the Weibull distribution under generalized Type-II hybrid censored data, deriving Bayesian estimators based on both symmetric and asymmetric loss functions. Li and Gui et al. [17] studied maximum likelihood estimation and Bayesian estimation of Shannon entropy for the Lomax distribution under generalized progressive hybrid censoring, employing Lindley’s approximation and the Tierney–Kadane method. Yu et al. [18] investigated statistical inference for the parameters and Shannon entropy of the inverse Weibull distribution under progressive first-failure censoring. Ren and Hu et al. [19] examined maximum likelihood estimation and Bayesian estimation of Shannon entropy and Rényi entropy for the two-parameter inverse Weibull distribution under the entropy loss function and the scaled squared error loss function.

Although the studies reviewed above have made important advances in entropy estimation under censored samples, the existing literature exhibits several notable gaps. Hassan et al. [16] investigated Bayesian estimation of entropy for the Weibull distribution under hybrid censoring; however, the distribution employed lacks the capacity to characterize non-monotonic failure rates afforded by the transmutation parameter of the TWD. Yu et al. [18] and Ren et al. [19] extended entropy inference for the inverse Weibull distribution to censored settings, yet neither addressed the construction of highest posterior density (HPD) credible intervals under the Progressive Type-II censoring scheme. Regarding the TWD specifically, Khan et al. [20] first derived the analytical expression for Shannon entropy of the TWD in the uncensored setting, establishing foundational properties including Rényi and q-entropies. Yousaf et al. [7], while conducting Bayesian estimation for the TWD, focused exclusively on the model parameters themselves rather than on Shannon entropy, which carries broader information-theoretic significance. In summary, no existing study simultaneously encompasses a systematic framework integrating maximum likelihood estimation, Bayesian point estimation under multiple loss functions, asymptotic confidence intervals (ACI), Bootstrap intervals, and HPD credible intervals for Shannon entropy of the TWD under Progressive Type-II censored samples. This gap constitutes the core motivation of the present work.

(1) Statistical inference for the Shannon entropy of the Transmuted Weibull Distribution based on Progressive Type-II censored samples has received relatively little attention in the existing literature. To fill this gap, this paper investigates statistical inference for the Shannon entropy of the Transmuted Weibull Distribution. Under the Progressive Type-II censoring scheme, a maximum likelihood estimation procedure for Shannon entropy is developed. The asymptotic variance is derived using the Delta method, and both asymptotic confidence intervals (ACI) and Bootstrap confidence intervals are constructed, providing frequentist inferential tools for quantifying the uncertainty associated with entropy estimation. The performance and practical applicability of the proposed methods are further evaluated through extensive Monte Carlo simulation studies.

(2) This paper develops a comprehensive Bayesian statistical inference framework for the Shannon entropy of the Transmuted Weibull Distribution based on Progressive Type-II censored samples. Extending the existing literature, we systematically investigate the construction of highest posterior density (HPD) credible intervals for Shannon entropy, together with Bayesian point estimators under three widely used loss functions. Under a joint prior specification consisting of Gamma and Uniform distributions, a hybrid Gibbs sampling algorithm is implemented within the Markov chain Monte Carlo (MCMC) framework to obtain posterior samples, from which the posterior mean, posterior median, and posterior mode are derived as Bayesian estimators of Shannon entropy. Under three representative censoring schemes, the maximum likelihood and Bayesian methods are systematically compared in terms of bias, mean square error (MSE), and confidence interval coverage probability. The results demonstrate that the Bayesian approach consistently outperforms the maximum likelihood method, particularly under small sample sizes and high censoring proportions. Finally, an empirical analysis based on a medical survival dataset further illustrates the feasibility and robustness of the proposed methods. These findings provide a practical inferential framework for entropy estimation under complex censoring schemes and offer effective Bayesian tools for the reliability assessment of highly reliable systems.

In summary, this paper presents a comprehensive statistical inferential framework for the Shannon entropy of the Transmuted Weibull Distribution under Progressive Type-II censored samples. The proposed framework integrates both maximum likelihood and Bayesian approaches for point and interval estimation, and is supported by a systematic performance comparison under a range of representative censoring schemes. The results demonstrate the effectiveness of the proposed methods and establish a practical inferential framework for uncertainty quantification of highly reliable systems under censored data. Furthermore, this study provides a useful reference for future research on entropy estimation and statistical inference in reliability analysis.

## 2. Prerequisite Knowledge and Theoretical Foundations

### 2.1. Transmuted Weibull Distribution

If a random variable X has the cumulative distribution function and probability density function given below, then X is said to follow a three-parameter Weibull distribution, denoted $X∼W\left(a,b,c\right)$, where a is the shape parameter, b is the scale parameter, and c is the location parameter.

(1) $$F_{W}\left(x;a,b,c\right)=1-\exp\left(-{\left(\frac{x-c}{b}\right)}^{a}\right)$$

(2) $$f_{W}\left(x;a,b,c\right)=\frac{a}{b}{\left(\frac{x-c}{b}\right)}^{a-1}\exp\left(-{\left(\frac{x-c}{b}\right)}^{a}\right)$$

When c=0, Equations (1) and (2) reduce to the CDF and PDF of the two-parameter Weibull distribution, respectively, in which case the random variable X follows a two-parameter Weibull distribution, denoted $X∼W\left(a,b\right)$. Its cumulative distribution function and probability density function are given, respectively, by

(3) $$G_{0}\left(x;a,b\right)=1-\exp\left(-{\left(\frac{x}{b}\right)}^{a}\right)$$

(4) $$g_{0}\left(x;a,b\right)=\frac{a}{b}{\left(\frac{x}{b}\right)}^{a-1}\exp\left(-{\left(\frac{x}{b}\right)}^{a}\right)$$

Shaw and Buckley et al. [21] proposed the quadratic rank transmutation map (QRTM) technique. If a random variable has the cumulative distribution function $F_{T}\left(x\right)$ and probability density function $f_{T}\left(x\right)$ as shown below, then X is said to follow a transmuted distribution:

(5) $$F_{T}\left(x;\lambda \right)=\left(1+\lambda \right)G\left(x\right)-\lambda G{\left(x\right)}^{2}$$

(6) $$f_{T}\left(x;\lambda \right)=g\left(x\right)\left[\left(1+\lambda \right)-2\lambda G\left(x\right)\right]$$

where λ is the transmutation parameter, and $G\left(x\right)$ and $g\left(x\right)$ are the cumulative distribution function and probability density function of the baseline distribution, respectively.

Based on the quadratic rank transmutation theory, any continuous distribution may serve as the baseline distribution. By substituting the functional expressions of the target distribution into $G_{0}\left(x\right)$ and $g_{0}\left(x\right)$ in Equations (5) and (6), the corresponding family of transmuted distributions can be constructed.

This paper transforms the two-parameter Weibull distribution as the base distribution to obtain the TWD. Its CDF $F\left(x\right)$ and PDF $f\left(x\right)$ are respectively, as follows:

(7) $$F\left(x;a,b,\lambda \right)=\left[1-\exp\left(-{\left(\frac{x}{b}\right)}^{a}\right)\right]\left[1+\lambda \exp\left(-{\left(\frac{x}{b}\right)}^{a}\right)\right]$$

(8) $$f\left(x;a,b,\lambda \right)=\frac{a}{b^{a}}x^{a-1}\exp\left(-{\left(\frac{x}{b}\right)}^{a}\right)\left[1-\lambda +2\lambda \exp\left(-{\left(\frac{x}{b}\right)}^{a}\right)\right]$$

where a>0,b>0 and −1≤λ≤1. A random variable X having the above CDF and PDF is said to follow the Transmuted Weibull Distribution TWD, denoted $W∼TW\left(a,b,\lambda \right)$.

**Figure 1:**
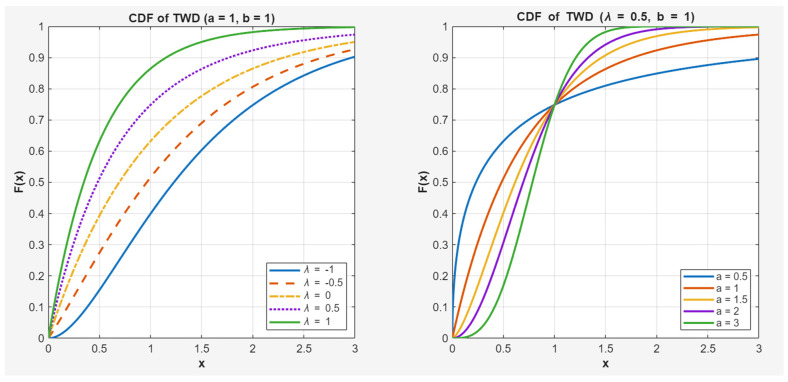
Cumulative distribution function of the Transmuted Weibull distribution.

**Figure 2:**
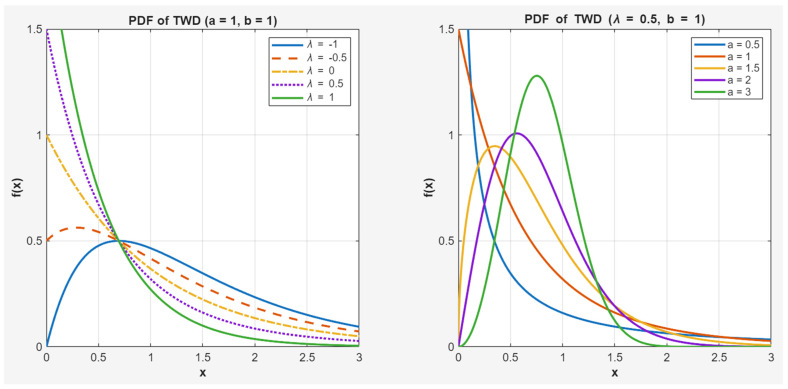
Probability density function of the Transmuted Weibull distribution.

It should be noted that the Transmuted Weibull Distribution is an extended model capable of analyzing more complex data, and it generalizes several widely used distributions. When a=1, the transmuted exponential distribution is obtained, as discussed in Shaw [9]; when λ=0, the TWD reduces to the standard Weibull distribution; and when a=λ=1, the resulting distribution is an exponential distribution with parameter $\frac{b}{2}$. Figure 1 illustrates the shape characteristics of the cumulative distribution function under various parameter combinations. Figure 2 displays the possible shapes of the probability density function of the TWD for selected values of λ and a with b=1 fixed. From a structural standpoint, λ functions as a shape-control parameter rather than a simple location or scale adjustment. Through the quadratic rank transmutation map in Equation (5), λ enters the cumulative distribution function as $F_{T}(x;\lambda )=(1+\lambda )G(x)-\lambda G{\left(x\right)}^{2}$, a quadratic—rather than linear—function of the baseline CDF $G\left(x\right)$. This quadratic dependence allows λ to redistribute probability mass nonlinearly across the support of X, in a way that simply adjusting the baseline Weibull’s shape or scale parameters cannot replicate. As illustrated in Figure 2, varying λ alone can shift the density from a monotonically decreasing form toward a peaked, unimodal shape, reflecting changes in skewness and kurtosis beyond what the two-parameter Weibull baseline can express. This added flexibility is particularly relevant for lifetime data exhibiting non-monotonic failure rates—such as the elevated early-life or wear-out hazards common in reliability and biomedical applications.

### 2.2. Definition of Shannon Entropy and Its Derivation Under TWD

For a continuous random variable X with probability density function f(x), the Shannon entropy H(x) is defined as

(9) H(f)=E(−lnf(x))

**Theorem** **1.** *If the random variable X follows the Transmuted Weibull Distribution, i.e., $X∼TW\left(a,b,\lambda \right)$, with probability density function $f\left(x;a,b,\lambda \right)$ as given in Equation (7), let $\theta =\left(a,b,\lambda \right)$; then, the Shannon entropy of the Transmuted Weibull Distribution is*

(10) $$\begin{array}{l} H(\theta )=E\left[-\ln f\left(x;a,b,\lambda \right)\right]\\ \;\;\;\;=-\ln(a)+\ln(b)-(a-1)E\left(\ln\frac{x}{b}\right)\\ \;\;\;\;+E[{\left(\frac{x}{b}\right)}^{a}]-E\left[\ln\left(1-\lambda +2\lambda e^{-{\left(x/b\right)}^{a}}\right)\right] \end{array}$$

The expected values in Equation (10) are obtained through the following steps:

$$E\left[\ln\left(\frac{x}{b}\right)\right]=\Gamma^{\left(\frac{1}{a}\right)}(1)-\lambda \ln{\left(2\right)}^{\frac{1}{a}}$$

$$E\left[\ln\left(1-\lambda +2\lambda e^{-{\left(\frac{x}{b}\right)}^{a}}\right)\right]=\ln(1-\lambda )+\sum_{k=1}^{\infty }\frac{{\left(-1\right)}^{k+1}}{k}{\left(\frac{2\lambda }{1-\lambda }\right)}^{k}\int_{0}^{\infty }e^{-k{\left(\frac{x}{b}\right)}^{a}}f(x;a,b,\lambda )dx$$

The formula for calculating the above points is

$$\begin{array}{l} K=\int_{0}^{\infty }\frac{a}{b}{\left(\frac{x}{b}\right)}^{a-1}e^{-(k+1){\left(\frac{x}{b}\right)}^{a}}\left[1-\lambda +2\lambda e^{-{\left(\frac{x}{b}\right)}^{a}}\right]dx\\ K=(1-\lambda )\int_{0}^{\infty }\frac{a}{b}{\left(\frac{x}{b}\right)}^{a-1}e^{-(k+1){\left(\frac{x}{b}\right)}^{a}}dx+2\lambda \int_{0}^{\infty }\frac{a}{b}{\left(\frac{x}{b}\right)}^{a-1}e^{-(k+2){\left(\frac{x}{b}\right)}^{a}}dx\\ K=\frac{1-\lambda }{k+1}+\frac{2\lambda }{k+2} \end{array}$$

The expected value in Equation (10) can be obtained by integration:

$$E\left[\ln\left(1-\lambda +2\lambda e^{-{\left(\frac{x}{b}\right)}^{a}}\right)\right]=\ln(1-\lambda )+\sum_{k=1}^{\infty }\frac{{\left(-1\right)}^{k+1}}{k!}{\left(\frac{2\lambda }{1-\lambda }\right)}^{k}\left(\frac{1-\lambda }{k+1}+\frac{2\lambda }{k+2}\right)$$

Finally, we obtain

(11) $$\begin{array}{l} H(\theta )=-\ln(a)+\ln(b)-(a-1)\left[\Gamma^{\left(\frac{1}{a}\right)}(1)-\lambda \ln{\left(2\right)}^{\left(\frac{1}{a}\right)}\right]+\\ \;\;\;\left(1-\frac{\lambda }{2}\right)-\ln(1-\lambda )-\sum_{k=1}^{\infty }\frac{{\left(-1\right)}^{k+1}}{k}{\left(\frac{2\lambda }{1-\lambda }\right)}^{k}\left(\frac{1-\lambda }{k+1}+\frac{2\lambda }{k+2}\right) \end{array}$$

**Remark.**  *The series expansion used in the derivation of Equation (11) requires $\left|\frac{2\lambda }{1-\lambda }e^{-{\left(\frac{x}{b}\right)}^{a}}\right|\leq 1$. Since $e^{-{\left(x/b\right)}^{a}}\in \left(0,\left.1\right]\right.$, this condition holds for all x>0 if and only if $\lambda \in \left[-1,1/3\right]$; the case λ=−1 poses no difficulty, since 1−λ=2≠0 there. For $\lambda \in \left(\left.1/3,1\right]\right.$, the denominator 1−λ becomes small (or vanishes at λ=1), the ratio $\left|\frac{2\lambda }{1-\lambda }\right|$ exceeds 1, and the series no longer converges. For $\lambda \in \left(1/3,\left.1\right]\right.$, Equation (11) is therefore not valid, and $H\left(\theta \right)$ should instead be evaluated by direct numerical integration of $E\left[\ln\left(1-\lambda +2\lambda e^{-{\left(x/b\right)}^{a}}\right)\right]$ rather than via the series expansion.*

## 3. Maximum Likelihood Estimation

### 3.1. Construction of the Joint Likelihood Function

Suppose $X_{1:m:n},X_{2:m:n},\ldots ,X_{m:m:n}$ are m PC-II samples observed from n test units, where each $X_{i:m:n}\left(0<i<m\right)$ follows $X∼TW\left(a,b,\lambda \right)$. The likelihood function of parameters a,b,λ under Progressive Type-II censored samples is

(12) $$L(a,b,\lambda |x)={\left(\frac{a}{b^{a}}\right)}^{m}\left[\prod_{i=1}^{m}x_{i}^{a-1}\right]e^{-\frac{1}{b^{a}}\sum_{i=1}^{m}\left(R_{i}+1\right)x_{i}^{a}}\prod_{i=1}^{m}\left[1-\lambda +2\lambda e^{-{\left(x_{i}/b\right)}^{a}}\right]$$

where $x=\left(x_{1:m:n},x_{2:m:n},\ldots ,x_{m:m:n}\right)$ is the observed failure data. The general likelihood under Progressive Type-II censoring includes a combinatorial constant $C=n\left(n-R_{1}-1\right)\left(n-R_{1}-R_{2}-1\right)\cdots \left(n-R_{1}-\cdots -R_{m-1}-m+1\right)$, counting the number of ways to select the censored units at each failure time. Since C does not depend on the parameters $\left(a,b,\lambda \right)$, it is omitted from Equation (13) without affecting the resulting maximum likelihood estimates.

The log-likelihood function is

(13) $$\begin{array}{l} \ln L(a,b,\lambda |x)=m(\ln a-a\ln b)+(a-1)\sum_{i=1}^{m}\ln x_{i}-\sum_{i=1}^{m}\left(1+R_{i}\right){\left(\frac{x_{i}}{b}\right)}^{a}\\ \;\;\;\;\;\;+\sum_{i=1}^{m}\ln\left[1-\lambda +2\lambda e^{-{\left(\frac{x_{i}}{b}\right)}^{a}}\right]+\sum_{i=1}^{m}R_{i}\ln\left[1-\lambda +\lambda e^{-{\left(\frac{x_{i}}{b}\right)}^{a}}\right] \end{array}$$

Maximum likelihood estimation has been widely applied in inference studies across various distributional models. Aggarwala [11] and Balakrishnan [22] constructed maximum likelihood estimators (MLE), uniformly minimum variance unbiased estimators, and best linear unbiased estimators for the two-parameter exponential distribution under Progressive Type-II censored samples, and derived the associated moment recurrence relations for computing variances and covariances. In the study of competing risks models for the Lomax distribution, researchers employed the PC-II mechanism to design optimal censoring schemes and investigated parameter estimation problems by maximizing the expected Fisher information [23]. Regarding the discrimination problem between the Weibull and log-normal distributions, Elsherpieny et al. [24] constructed discrimination statistics using maximum likelihood function values under progressive censoring and conducted numerical simulations of the correct selection probability under small sample conditions.

### 3.2. Parameter Estimation Methods

Under Progressive Type-II censored samples, this paper first constructs the log-likelihood function for the parameters of the TWD, and derives the maximum likelihood estimation equations with respect to the shape parameter a, scale parameter b, and transmutation parameter λ. The specific steps are as follows.

For notational convenience, we introduce the following simplifications:

Let $A_{i}={\left(\frac{x_{i}}{b}\right)}^{a}$

Let $E_{i}=e^{-A_{i}}=e^{-{\left(\frac{x_{i}}{b}\right)}^{a}}$

Let $Y_{i}=\ln\left(\frac{x_{i}}{b}\right)$

Taking the partial derivative of the log-likelihood function with respect to the shape parameter a:


(14) $$\frac{\partial \ln L}{\partial a}=\frac{\partial \ln L}{\partial a}=\frac{m}{a}+\sum_{i=1}^{m}Y_{i}\left[1-A_{i}-R_{i}A_{i}-\frac{2\lambda E_{i}A_{i}}{1-\lambda +2\lambda E_{i}}-\frac{R_{i}\lambda E_{i}A_{i}}{1-\lambda +\lambda E_{i}}\right]$$

Taking the partial derivative with respect to the scale parameter b:


(15) $$\frac{\partial \ln L}{\partial b}=\frac{a}{b}\left(-m+\sum_{i=1}^{m}\left[A_{i}+R_{i}A_{i}+\frac{2\lambda E_{i}A_{i}}{1-\lambda +2\lambda E_{i}}+\frac{R_{i}\lambda E_{i}A_{i}}{1-\lambda +\lambda E_{i}}\right]\right)$$

Taking the partial derivative with respect to the transmutation parameter λ:


(16) $$\frac{\partial \ln L}{\partial \lambda }=\sum_{i=1}^{m}\frac{2E_{i}-1}{1-\lambda +2\lambda E_{i}}+\sum_{i=1}^{m}\frac{R_{i}(E_{i}-1)}{1-\lambda +\lambda E_{i}}$$

The solution to equation system (17) yields the maximum likelihood estimators of the parameters. Since this system of equations generally has no closed-form solution, numerical optimization algorithms such as the Newton–Raphson method are employed to solve it, yielding the estimates $\hat{a},\hat{b},\hat{\lambda }$.

(17) $$\left\{\begin{array}{c} \frac{\partial \ln L}{\partial a}=0\\ \frac{\partial \ln L}{\partial b}=0\\ \frac{\partial \ln L}{\partial \lambda }=0 \end{array}\right\}$$

The Newton–Raphson method is then applied to obtain the maximum likelihood estimates of a,b,λ Assuming the parameter vector $\theta ={\left(a,b,\lambda \right)}^{T}$ has maximum likelihood estimator $\hat{\theta }={\left(\hat{a},\hat{b},\hat{\lambda }\right)}^{T}$ the procedure is as follows:

(I) Specify the likelihood function and take its logarithm to obtain the log-likelihood function.
(II) Compute the first-order and second-order derivatives of the log-likelihood function.
(III) Initialize the parameter values, typically using the sample mean or other empirical values as starting values.
(IV) Compute the first- and second-order derivatives at the current parameter values.
(V) Update the parameter values using the Newton–Raphson iteration formula.
  (18) $${\hat{\theta }}_{new}={\hat{\theta }}_{old}-Hess{\left({\hat{\theta }}_{old}\right)}^{-1}\nabla \ln L\left({\hat{\theta }}_{old}\right)$$
  where $\nabla \ln L\left(\theta \right)$ denotes the vector of first-order partial derivatives of the log-likelihood function with respect to θ, i.e., the gradient vector. Let $Hess\left(\theta \right)$ denote the Hessian matrix of the log-likelihood function with respect to θ, and Hess $Hess{\left(\theta \right)}^{-1}$ its inverse.
(VI) Repeat steps (IV) and (V) until the parameter values converge or the prescribed number of iterations is reached. The final parameter values are the maximum likelihood estimates.

On this basis, by virtue of the invariance property of maximum likelihood estimators, there is no significant discrepancy among the parameter estimates obtained during the iterative process. Accordingly, the estimates $\hat{a},\hat{b},\hat{\lambda }$ are substituted for a,b,λ in Equation (11). For ease of exposition, the maximum likelihood estimator of Shannon entropy is denoted ${\hat{H}}_{ML}$, whose expression is given as follows:

(19) $$\begin{array}{cc} {\hat{H}}_{ML} & =-\ln\left(\hat{a}\right)+\ln\left(\hat{b}\right)-\left(\hat{a}-1\right)\left[\Gamma^{\left(\frac{1}{\hat{a}}\right)}\left(1\right)-\hat{\lambda }\ln{\left(2\right)}^{\left(\frac{1}{\hat{a}}\right)}\right]\\ & +\left(1-\frac{\hat{\lambda }}{2}\right)-\ln\left(1-\hat{\lambda }\right)-\sum_{k=1}^{\infty }\frac{{\left(-1\right)}^{k+1}}{k}{\left(\frac{2\hat{\lambda }}{1-\hat{\lambda }}\right)}^{k}\left(\frac{1-\hat{\lambda }}{k+1}+\frac{2\hat{\lambda }}{k+2}\right) \end{array}$$

To enforce the strict positivity of the parameters during numerical optimization, we maximize the log-likelihood function over an unconstrained, reparameterized space. Specifically, by defining a=lna and b=lnb, the parameter constraints a>0 and b>0 are automatically satisfied at each iteration step; this closely mirrors the log-space proposal construction already adopted for a and b in the MCMC sampler of Section 4.2. For the shape parameter lambda, the boundary constraint lambda in (−1,1) is structurally managed via an appropriate logit-type transformation to ensure the iterative trajectory remains within the admissible domain. The Newton–Raphson updates are subsequently executed on the transformed parameter vector via the chain rule. Upon mapping the final iterations back to the original scales, the resulting estimates $\left(\hat{a},\hat{b},\hat{\lambda }\right)$ are mathematically guaranteed to lie within the admissible domain $\left(0,\infty \right)\times \left(0,\infty \right)\times \left(-1,1\right)$.

### 3.3. Existence and Uniqueness of the Maximum Likelihood Estimators

**Theorem** **2.** *Let $x_{1}<x_{2}<\cdots <x_{m}$ be a Progressive Type-II censored sample from the Transmuted Weibull Distribution $TWD\left(a,b,\lambda \right)$, where $a>0,b>0,\lambda \in \left[-1,1\right]$. For any fixed values of the other two parameters, the log-likelihood $\ln L\left(a,b,\lambda \right)$ is strictly concave along each individual coordinate direction, and the corresponding likelihood equation has a unique root on its domain.*

**Proof.**  Denote the log-likelihood function as $L\left(a,b,\lambda \right)$, and set its partial derivatives with respect to each of the three parameters equal to zero to obtain the likelihood Equation (17). The existence and uniqueness of the roots of these equations are discussed separately for each parameter. □

#### 3.3.1. Existence and Uniqueness for Parameter a

$\frac{\partial L}{\partial a}$ is a continuous function on $a\in \left(0,+\infty \right)$. Its limiting behavior at the boundaries is analyzed as follows:

As $a\to 0^{+}$, the log-likelihood equation contains the term $\frac{m}{a}\to +\infty$, while the remaining terms remain bounded at this limit. Therefore,

(20) $$\underset{a\to 0^{+}}{\lim}\frac{\partial L}{\partial a}=+\infty$$

As a→+∞, for observations satisfying $x_{i}\neq b$, the term $A_{i}\ln\left(\frac{x_{i}}{b}\right)\to -\infty$, and therefore

(21) $$\underset{a\to +\infty }{\lim}\frac{\partial L}{\partial a}=-\infty$$

By the intermediate value theorem for continuous functions, the equation $\frac{\partial L}{\partial a}=0$ has at least one root on $\left(0,+\infty \right)$, i.e., $\hat{a}$ exists.

Regarding uniqueness, from Equation (27) in Section 3.4, it follows that $-\frac{m}{a^{2}}<0$ holds identically, while all remaining terms are ratios of complete square terms with negative numerators to positive denominators. Consequently, $\frac{\partial^{2}L}{\partial a^{2}}<0$ holds identically throughout the parameter domain, meaning that $\frac{\partial L}{\partial a}$ is strictly monotonically decreasing with respect to a, and hence the equation has a unique root. Therefore, for any fixed $\left(b,\lambda \right)$, lnL is strictly concave in a on $\left(0,\infty \right)$, and $\hat{a}\left(b,\lambda \right)$ exists and is the unique maximizer along this coordinate direction.

#### 3.3.2. Existence and Uniqueness for Parameter b

$\frac{\partial L}{\partial b}$ is a continuous function on $b\in \left(0,+\infty \right)$. Its limiting behavior at the boundaries is analyzed as follows:

As $b\to 0^{+}$, ${\left(\frac{x_{i}}{b}\right)}^{a}\to +\infty$, the reliability function tends to 0, and the positive terms in the log-likelihood equation associated with the reliability function dominate the whole expression. Therefore,

(22) $$\underset{b\to 0^{+}}{\lim}\frac{\partial L}{\partial b}=+\infty$$

As b→+∞, ${\left(\frac{x_{i}}{b}\right)}^{a}\to 0$, the reliability function tends to 1, and the negative term containing $\frac{m}{b}$ dominates. Therefore,

(23) $$\underset{b\to +\infty }{\lim}\frac{\partial L}{\partial b}=-\infty$$

By the intermediate value theorem, $\hat{b}$ exists. From Equation (31) in Section 3.4, all terms carry negative signs with positive denominators, so $\frac{\partial^{2}L}{\partial b^{2}}<0$ holds identically throughout the entire parameter domain, meaning that $\frac{\partial L}{\partial b}$ is strictly monotonically decreasing with respect to b, and hence the equation has a unique root. Therefore, for any fixed $\left(a,\lambda \right)$, lnL is strictly concave in a on $\left(0,\infty \right)$, and $\hat{b}\left(a,\lambda \right)$ exists and is the unique maximizer along this coordinate direction.

#### 3.3.3. Existence and Uniqueness for Parameter λ

$\frac{\partial L}{\partial \lambda }$ is a continuous function on $\lambda \in \left[-1,1\right]$. Its sign at the boundary points is analyzed as follows:

As $\lambda \to -1^{+}$, the positive terms in the log-likelihood equation associated with $1-\lambda +2\lambda E_{i}$ dominate. Therefore,

(24) $${\left.\frac{\partial L}{\partial \lambda }\right|}_{\lambda =-1^{+}}>0$$

As $\lambda \to 1^{-}$, the sign of the above terms reverses and the negative terms dominate. Therefore:

(25) $${\left.\frac{\partial L}{\partial \lambda }\right|}_{\lambda =1^{-}}<0$$

By the intermediate value theorem, $\hat{\lambda }$ exists. From Equation (33) in Section 3.4, the numerator of each term is the square of a real number with a negative sign in front, and the denominators remain positive throughout the domain, so $\frac{\partial L}{\partial \lambda }$ is strictly monotonically decreasing with respect to λ and hence the equation has a unique root. Therefore, for any fixed $\left(a,b\right)$, lnL is strictly concave in λ on $\left(-1,1\right)$, and $\hat{\lambda }\left(a,b\right)$ exists and is the unique maximizer along this coordinate direction.

In summary, the likelihood equation system (17) is strictly concave along each individual coordinate axis, which guarantees that the coordinate-wise updates in the Newton–Raphson procedure of Section 3.2 are well-defined and that each one-dimensional update step does not decrease the log-likelihood. This coordinate-wise concavity, however, does not by itself imply joint concavity of $\ln L\left(a,b,\lambda \right)$ over the full three-dimensional parameter space: a function may be concave along each coordinate direction individually while still failing to be jointly concave, as is consistent with the sign-indeterminate mixed partial derivatives in Equations (27)–(35). We therefore do not claim a proof of global joint uniqueness of the maximum likelihood estimator $\hat{\theta }=\left(\hat{a},\hat{b},\hat{\lambda }\right)$. The existence of a stationary point follows from the continuity of lnL together with the coordinate-wise results above; global uniqueness is instead supported numerically—across all Monte Carlo replications in Section 5, Newton–Raphson initialized from several distinct starting values converged to the same solution in every case.

### 3.4. Asymptotic Confidence Intervals

To quantify the uncertainty associated with entropy estimation, this section constructs asymptotic confidence intervals (ACI) based on large-sample asymptotic theory. The construction of the ACI is based on the score vector obtained by differentiating the log-likelihood function with respect to the parameter vector theta, while the asymptotic variance–covariance matrix of the maximum likelihood estimators is derived from the Fisher information matrix. The asymptotic variance of the Shannon entropy estimator is subsequently obtained using the Delta method, based on which the corresponding asymptotic confidence intervals are constructed.

The Fisher information matrix I(θ) is defined as the matrix formed by the negative expected values of the second-order partial derivatives of the log-likelihood function, and its inverse matrix is the asymptotic covariance matrix of the parameter estimators.

(26) $$I\left(\theta \right)=-E\left[\begin{array}{ccc} \frac{\partial^{2}\ln L}{\partial a^{2}} & \frac{\partial^{2}\ln L}{\partial a\partial b} & \frac{\partial^{2}\ln L}{\partial a\partial \lambda }\\ \frac{\partial^{2}\ln L}{\partial b\partial a} & \frac{\partial^{2}\ln L}{\partial b^{2}} & \frac{\partial^{2}\ln L}{\partial b\partial \lambda }\\ \frac{\partial^{2}\ln L}{\partial \lambda \partial a} & \frac{\partial^{2}\ln L}{\partial \lambda \partial b} & \frac{\partial^{2}\ln L}{\partial \lambda^{2}} \end{array}\right]$$

where the individual elements are

(27) $$\begin{array}{l} \frac{\partial^{2}\ln L}{\partial a^{2}}=-\frac{m}{a^{2}}-\sum_{i=1}^{m}A_{i}Y_{i}^{2}\left(1+R_{i}\right)-\\ \;\;\;\;\sum_{i=1}^{m}\frac{4\lambda^{2}E_{i}^{2}A_{i}^{2}Y_{i}^{2}}{{\left(1-\lambda +2\lambda E_{i}\right)}^{2}}-\sum_{i=1}^{m}\frac{R_{i}\lambda^{2}E_{i}^{2}A_{i}^{2}Y_{i}^{2}}{{\left(1-\lambda +\lambda E_{i}\right)}^{2}} \end{array}$$

(28) $$\begin{array}{l} \frac{\partial^{2}\ln L}{\partial a\partial b}=\frac{a}{b}\sum_{i=1}^{m}\left(1+R_{i}\right)A_{i}Y_{i}-\sum_{i=1}^{m}\frac{2\lambda aE_{i}A_{i}Y_{i}}{b(1-\lambda +2\lambda E_{i})}\\ \;\;\;\;\;+\sum_{i=1}^{m}\frac{2\lambda a(1-\lambda )E_{i}A_{i}Y_{i}}{b{\left(1-\lambda +2\lambda E_{i}\right)}^{2}}-\sum_{i=1}^{m}\frac{4\lambda^{2}aE_{i}^{2}A_{i}^{2}Y_{i}}{b{\left(1-\lambda +2\lambda E_{i}\right)}^{2}}\\ \;\;\;\;\;-\sum_{i=1}^{m}\frac{R_{i}\lambda aE_{i}A_{i}Y_{i}}{b(1-\lambda +\lambda E_{i})}+\sum_{i=1}^{m}\frac{R_{i}\lambda a(1-\lambda )E_{i}A_{i}Y_{i}}{b{\left(1-\lambda +\lambda E_{i}\right)}^{2}}\\ \;\;\;\;-\sum_{i=1}^{m}\frac{R_{i}\lambda^{2}aE_{i}^{2}A_{i}^{2}Y_{i}}{b{\left(1-\lambda +\lambda E_{i}\right)}^{2}} \end{array}$$

(29) $$\frac{\partial^{2}\ln L}{\partial a\partial \lambda }=-\sum_{i=1}^{m}\frac{2E_{i}A_{i}Y_{i}}{{\left(1-\lambda +2\lambda E_{i}\right)}^{2}}-\sum_{i=1}^{m}\frac{R_{i}E_{i}A_{i}Y_{i}}{{\left(1-\lambda +\lambda E_{i}\right)}^{2}}$$

(30) $$\begin{array}{l} \frac{\partial^{2}\ln L}{\partial b\partial a}=\frac{a}{b}\sum_{i=1}^{m}\left(1+R_{i}\right)A_{i}Y_{i}-\sum_{i=1}^{m}\frac{2\lambda aE_{i}A_{i}Y_{i}}{b(1-\lambda +2\lambda E_{i})}\\ \;\;\;\;\;\;+\sum_{i=1}^{m}\frac{2\lambda a(1-\lambda )E_{i}A_{i}Y_{i}}{b{\left(1-\lambda +2\lambda E_{i}\right)}^{2}}-\sum_{i=1}^{m}\frac{4\lambda^{2}aE_{i}^{2}A_{i}^{2}Y_{i}}{b{\left(1-\lambda +2\lambda E_{i}\right)}^{2}}\\ \;\;\;\;\;\;-\sum_{i=1}^{m}\frac{R_{i}\lambda aE_{i}A_{i}Y_{i}}{b(1-\lambda +\lambda E_{i})}+\sum_{i=1}^{m}\frac{R_{i}\lambda a(1-\lambda )E_{i}A_{i}Y_{i}}{b{\left(1-\lambda +\lambda E_{i}\right)}^{2}}\\ \;\;\;\;\;-\sum_{i=1}^{m}\frac{R_{i}\lambda^{2}aE_{i}^{2}A_{i}^{2}Y_{i}}{b{\left(1-\lambda +\lambda E_{i}\right)}^{2}} \end{array}$$

(31) $$\begin{array}{l} \frac{\partial^{2}\ln L}{\partial b^{2}}=\frac{ma}{b^{2}}-\sum_{i=1}^{m}\frac{a(1+a)(1+R_{i})A_{i}}{b^{2}}+\sum_{i=1}^{m}\frac{2\lambda a^{2}E_{i}A_{i}}{b^{2}(1-\lambda +2\lambda E_{i})}\\ \;\;\;\;\;\;-\sum_{i=1}^{m}\frac{4\lambda^{2}a^{2}E_{i}^{2}A_{i}^{2}}{b^{2}{\left(1-\lambda +2\lambda E_{i}\right)}^{2}}+\sum_{i=1}^{m}\frac{R_{i}\lambda a^{2}E_{i}A_{i}}{b^{2}(1-\lambda +\lambda E_{i})}\\ \;\;\;\;\;\;-\sum_{i=1}^{m}\frac{R_{i}\lambda^{2}a^{2}E_{i}^{2}A_{i}^{2}}{b^{2}{\left(1-\lambda +\lambda E_{i}\right)}^{2}} \end{array}$$

(32) $$\frac{\partial^{2}\ln L}{\partial b\partial \lambda }=\frac{a}{b}\sum_{i=1}^{m}\frac{2A_{i}E_{i}}{{\left(1-\lambda +2\lambda E_{i}\right)}^{2}}+\frac{a}{b}\sum_{i=1}^{m}\frac{R_{i}A_{i}E_{i}}{\left(1-\lambda +\lambda E_{i}\right)}$$

(33) $$\frac{\partial^{2}\ln L}{\partial \lambda^{2}}=-\sum_{i=1}^{m}\frac{{\left(2E_{i}-1\right)}^{2}}{{\left(1-\lambda +2\lambda E_{i}\right)}^{2}}-\sum_{i=1}^{m}\frac{R_{i}{\left(E_{i}-1\right)}^{2}}{{\left(1-\lambda +\lambda E_{i}\right)}^{2}}$$

(34) $$\frac{\partial^{2}\ln L}{\partial \lambda \partial a}=-\sum_{i=1}^{m}\frac{2E_{i}A_{i}Y_{i}}{{\left(1-\lambda +2\lambda E_{i}\right)}^{2}}-\sum_{i=1}^{m}\frac{R_{i}E_{i}A_{i}Y_{i}}{{\left(1-\lambda +\lambda E_{i}\right)}^{2}}$$

(35) $$\frac{\partial^{2}\ln L}{\partial \lambda \partial b}=\frac{a}{b}\sum_{i=1}^{m}\frac{2A_{i}E_{i}}{{\left(1-\lambda +2\lambda E_{i}\right)}^{2}}+\frac{a}{b}\sum_{i=1}^{m}\frac{R_{i}A_{i}E_{i}}{\left(1-\lambda +\lambda E_{i}\right)}$$

Since the expectations in the Fisher information matrix are difficult to compute directly, the observed Fisher information matrix is typically used instead. The Fisher matrix can be written as

$$I\left(\hat{\theta }\right)=-{\left[\begin{array}{ccc} \frac{\partial^{2}\ln L}{\partial a^{2}} & \frac{\partial^{2}\ln L}{\partial a\partial b} & \frac{\partial^{2}\ln L}{\partial a\partial \lambda }\\ \frac{\partial^{2}\ln L}{\partial b\partial a} & \frac{\partial^{2}\ln L}{\partial b^{2}} & \frac{\partial^{2}\ln L}{\partial b\partial \lambda }\\ \frac{\partial^{2}\ln L}{\partial \lambda \partial a} & \frac{\partial^{2}\ln L}{\partial \lambda \partial b} & \frac{\partial^{2}\ln L}{\partial \lambda^{2}} \end{array}\right]}_{\theta =\hat{\theta }}$$

In this case, the inverse Fisher matrix $I^{-1}\left(\hat{\theta }\right)$ of the estimator $\hat{\theta }$ can be used directly as the inverse of the observed Fisher matrix $I\left(\hat{\theta }\right)$, namely

$$I^{-1}\left(\hat{\theta }\right)=\left[\begin{array}{ccc} Var\left(\hat{a}\right) & Cov\left(\hat{a},\hat{b}\right) & Cov\left(\hat{a},\hat{\lambda }\right)\\ Cov\left(\hat{b},\hat{a}\right) & Var\left(\hat{b}\right) & Cov\left(\hat{b},\hat{\lambda }\right)\\ Cov\left(\hat{\lambda },\hat{a}\right) & Cov\left(\hat{\lambda },\hat{b}\right) & Var\left(\hat{\lambda }\right) \end{array}\right]$$

where $Var\left(\cdot \right)$ denotes the variance of an estimator, and $Cov\left(\cdot ,\cdot \right)$ denotes the covariance between two estimators.

To construct an estimate of the asymptotic covariance matrix, the partial derivatives of the entropy function H(θ) with respect to each parameter are required. Define the gradient vector k(θ) as

$$k(\theta )=\nabla H(\theta )={\left(\frac{\partial H(\theta )}{\partial a},\frac{\partial H(\theta )}{\partial b},\frac{\partial H(\theta )}{\partial \lambda }\right)}^{T}$$

Substituting the maximum likelihood estimators yields $\hat{k}=k(\hat{\theta })$. By the Delta method, the asymptotic variance of the maximum likelihood estimator ${\hat{H}}_{ML}$ of Shannon entropy is estimated as

$$Var({\hat{H}}_{ML})={\hat{k}}^{T}I^{-1}(\hat{\theta })\hat{k}$$

Through the above derivation, the asymptotic variance estimate of the entropy is obtained, from which an asymptotic confidence interval in standard form can be constructed. The $100\left(1-\alpha \right)\%$ asymptotic confidence interval for Shannon entropy is

(36) $$\left[{\hat{H}}_{ML}-Z_{1-\alpha /2}\sqrt{Var({\hat{H}}_{ML})},{\hat{H}}_{ML}+Z_{1-\alpha /2}\sqrt{Var({\hat{H}}_{ML})}\right]$$

where $Z_{1-\alpha /2}$ is the (1−α/2) quantile of the standard normal distribution. This interval represents the range within which the true value of Shannon entropy may lie at a given confidence level 1−α.

### 3.5. Bootstrap Confidence Interval

The Bootstrap method is a resampling-based statistical inference technique proposed by Efron [25] in 1979. Its core idea is to generate a large number of Bootstrap samples by repeatedly drawing with replacement from the original sample, thereby constructing the empirical distribution of the statistic. This method does not rely on large-sample asymptotic theory, nor does it require strict parametric assumptions about the model distribution; consequently, it tends to provide more stable confidence interval estimates in small-sample settings or when the assumption of asymptotic normality is difficult to satisfy. Compared with traditional normal approximation-based methods, the Bootstrap method more accurately characterizes the actual sampling distribution of a statistic, and is particularly suited to situations where the distributional shape is complex, skewed, or heavy-tailed. Especially when handling Progressive Type-II censored data, the resampling procedure must preserve the original censoring structure to ensure that Bootstrap samples accurately reflect the informational characteristics of the original data, thereby providing a flexible and practical solution for interval estimation in complex censoring scenarios.

Accordingly, this section employs the Bootstrap method to construct Bootstrap confidence intervals for Shannon entropy. The specific steps are as follows:

(1) From the original sample $x=(x_{1},x_{2},\ldots ,x_{n})$ of size n, randomly draw n data points with replacement to generate a Bootstrap sample $x_{1}^{*},x_{2}^{*},x_{3}^{*},\ldots ,x_{n}^{*}$.
(2) Compute the maximum likelihood estimate of Shannon entropy based on the generated Bootstrap sample, denoted ${\hat{H}}^{*}$.
(3) Repeat the above steps N times to obtain N Shannon maximum likelihood estimates of Shannon entropy; arrange these estimates in ascending order and denote them ${\hat{H}}_{1}^{*},{\hat{H}}_{2}^{*},{\hat{H}}_{3}^{*},\ldots ,{\hat{H}}_{N}^{*}$.
(4) For a given confidence level 1−α, the Bootstrap confidence interval for Shannon entropy is given by

(37) $$\left[{\hat{H}}_{\left(\left\lceil N\cdot \alpha /2\right\rceil \right)}^{*},{\hat{H}}_{\left(\left\lfloor N\cdot \left(1-\alpha /2\right)\right\rfloor \right)}^{*}\right]$$

where $\left\lceil \cdot \right\rceil$ denotes the ceiling function,$\left\lfloor \cdot \right\rfloor$ denotes the floor function, and ${\hat{H}}_{i}^{*}$ (i=1,2,…,N) denotes the sorted Bootstrap entropy estimates.

## 4. Bayesian Inference

In reliability analysis and entropy estimation, maximum likelihood estimation is widely used because of its straightforward implementation. However, its performance may deteriorate considerably in the presence of small sample sizes or heavy censoring, resulting in substantial estimation bias and numerical instability. To address these limitations, this paper adopts a Bayesian inference framework. Within the Bayesian framework, model parameters are treated as random variables, and posterior inference is obtained by combining prior distributions with the likelihood function through Bayes’ theorem. Compared with maximum likelihood estimation, the Bayesian approach effectively incorporates prior information, thereby improving estimation accuracy, particularly in small-sample and high-censoring settings. Moreover, Bayesian inference yields the entire posterior distribution of the parameters, providing a more comprehensive characterization of estimation uncertainty. Correspondingly, credible intervals admit a direct probabilistic interpretation, representing the posterior probability that the parameter lies within a specified interval. Finally, when the posterior distribution does not possess a closed-form expression, efficient posterior inference can be carried out using numerical techniques such as Markov Chain Monte Carlo (MCMC).

In recent years, the Bayesian method has demonstrated broad applicability in reliability modeling and lifetime data analysis, providing stable and effective solutions for statistical inference under complex censoring conditions. Kundu and Pradhan [26] applied Bayesian inference to estimate the parameters of the Weibull distribution for Progressive Type-II competing risks censored data, computing Bayesian estimators and HPD credible intervals via MCMC techniques; Monte Carlo simulation results demonstrated that the method effectively handles complex posterior distributions. Balakrishnan and Mitra [27] studied Bayesian inference for the Weibull distribution under left-truncated and right-censored data, comparing the EM algorithm and the Newton–Raphson method; extensive Monte Carlo simulation showed that both methods yield very close results, validating the effectiveness of the estimation approach. Wang and Gui [28] investigated the estimation of Shannon entropy for the Burr Type XII distribution under progressive Type-II censoring. They developed both maximum likelihood and Bayesian estimation methods, constructed asymptotic confidence intervals and Bayesian credible intervals, and demonstrated through simulation studies that Bayesian estimators generally exhibited superior performance in terms of estimation accuracy under progressive censoring. Alshenawy et al. [29] conducted Bayesian inference for Shannon entropy of the Maxwell distribution based on progressive first-failure censored data, computing Bayesian estimators under different loss functions using the Tierney–Kadane approximation and MCMC methods; simulation results indicate that the Bayesian approach exhibits better estimation accuracy in small-sample settings. Mondal and Kundu [30] performed a comprehensive Bayesian analysis of the Weibull distribution under a balanced joint Type-II progressive censoring scheme; numerical results show that the Bayesian method outperforms maximum likelihood estimation in both mean square error and interval estimation. These studies collectively demonstrate the advantages of the Bayesian framework in handling complex censored data and uncertainty quantification, providing a solid theoretical foundation and methodological support for the approach proposed in this paper.

Against this background, this paper constructs a Bayesian inference framework for the TWD under Progressive Type-II censored samples, derives the posterior distributions of the parameters and the posterior expectation of entropy, and obtains the corresponding Bayesian estimators and credible intervals by combining multiple loss functions with numerical algorithms.

### 4.1. Prior and Posterior Distributions

In Bayesian inference, the prior distribution is combined with the likelihood function through Bayes’ theorem to obtain the posterior distribution, which forms the basis for statistical inference. An appropriate prior distribution effectively incorporates existing knowledge while allowing the sample information to dominate the posterior inference, thereby improving estimation accuracy, particularly in small-sample or high-censoring settings. According to the amount of prior information incorporated, prior distributions are commonly classified as informative, weakly informative, or non-informative. The choice of prior distribution should balance the incorporation of prior knowledge with the need to avoid introducing excessive subjective influence.

This paper adopts different prior distributions according to the characteristics of the model parameters. Specifically, independent Gamma prior distributions are assigned to the shape parameter and the scale parameter. The Gamma distribution is a natural choice for positive-valued parameters because its support is the positive real line, which is fully consistent with the parameter space of the Weibull Distribution. Furthermore, its shape can be flexibly controlled through the hyperparameters, allowing different levels of prior information to be incorporated. Owing to these desirable properties, the Gamma distribution has been widely adopted in Bayesian inference for censored lifetime data and has been shown to yield reliable posterior inference. For the transmutation parameter λ, a uniform prior is assumed; the uniform prior belongs to the class of non-informative priors, assigning equal probability to all values in the parameter space, thereby avoiding subjective preference and allowing the posterior inference to be driven primarily by the data.

This paper assumes a gamma distribution as the prior for a, denoted $p\left(a\right)$, with probability density function:

(38) $$p\left(a\right)=\frac{b_{0}^{a_{0}}}{\Gamma \left(a_{0}\right)}a^{a_{0}-1}e^{-b_{0}a},a>0$$

where, $a_{0}>0$ is the shape hyperparameter and $b_{0}>0$ is the rate hyperparameter.

Similarly, this paper assumes a gamma distribution as the prior for b, denoted $p\left(b\right)$, with probability density function:

(39) $$p\left(b\right)=\frac{d_{0}^{c_{0}}}{\Gamma \left(c_{0}\right)}b^{c_{0}-1}e^{-d_{0}b},b>0$$

where, $c_{0}>0$ is the shape hyperparameter and $d_{0}>0$ is the rate hyperparameter.

For the transmutation parameter λ, a uniform distribution U[−1,1] is assumed as the prior distribution; this choice indicates that every value of λ in the interval $\left[-1,1\right]$ is equally probable.

(40) $$p\left(\lambda \right)=\frac{1}{2},-1\leq \lambda \leq 1$$

The joint prior distribution is

(41) $$p\left(\theta \right)=\left(\frac{b_{0}^{a_{0}}}{\Gamma \left(a_{0}\right)}a^{a_{0}-1}e^{-b_{0}a}\right)\cdot \left(\frac{d_{0}^{c_{0}}}{\Gamma \left(c_{0}\right)}b^{c_{0}-1}e^{-d_{0}b}\right)\cdot \left(\frac{1}{2}\right)$$

where a>0,b>0,−1≤λ≤1.

Let $X_{1:m:n},X_{2:m:n},\ldots ,X_{m:m:n}$ be the m ordered failure observations obtained from a Progressive Type-II censoring experiment, where each observation $X_{1:m:n},X_{2:m:n},\ldots ,X_{m:m:n}$ follows the Transmuted Weibull Distribution, with probability density function given by Equation (6). Within the Bayesian framework, the joint posterior distribution of the parameter vector θ is obtained from the prior distribution and the likelihood function via Bayes’ theorem. The joint posterior distribution of the parameters is

(42) $$\frac{p(a)\cdot p(b)\cdot p(\lambda )\cdot L(\theta \left|x\right.)}{\int \int \int p(a)\cdot p(b)\cdot p(\lambda )\cdot L(\theta \left|x\right.)dadbd\lambda }$$

Substituting the prior distributions (38), (39),(40) and the likelihood function (12) into the above expression, we obtain

(43) $$\begin{array}{l} p\left(\theta |x\right)=\left[\prod_{i=1}^{m}\left(\frac{a}{b^{a}}x_{i}^{a-1}e^{-{\left(\frac{x_{i}}{b}\right)}^{a}}\left(1-\lambda +2\lambda e^{-{\left(\frac{x_{i}}{b}\right)}^{a}}\right)\left(1-\left(1-e^{-{\left(\frac{x_{i}}{b}\right)}^{a}}\right)\right.\right.\right.\\ \;\;\;\;\;\left.\left.{\left.\left(1+\lambda e^{-{\left(\frac{x_{i}}{b}\right)}^{a}}\right)\right)}^{R_{i}}\right)\right]\cdot \left[a^{a_{0}-1}e^{-b_{0}a}\right]\cdot \left[b^{c_{0}-1}e^{-d_{0}b}\right] \end{array}$$

In the Bayesian framework, parameter estimation depends on both the posterior distribution and the selected loss function. The loss function measures the discrepancy between an estimate and the true parameter value, and different loss functions lead to different optimal Bayesian estimators. To evaluate the performance and robustness of the proposed Bayesian procedure under different decision-theoretic criteria, this paper considers three commonly used loss functions for comparison, thereby providing a more comprehensive assessment of the estimation results.

- Squared Error Loss Function

The squared error loss function (SEL) is one of the most commonly used loss functions; it assigns greater penalty to larger deviations. Its form is

$$L\left(\hat{H},H\right)={\left(\hat{H}-H\right)}^{2}$$

Based on the squared error loss function, the Bayesian estimator ${\hat{H}}_{SEL}$ of Shannon entropy H(θ) is given by the following formula, where $E[\left.H(\theta )\right|x]$ denotes the posterior mean of H(θ):

(44) $${\hat{H}}_{SEL}=E[H(\theta )\left|x\right.]=\int_{0}^{+\infty }\int_{0}^{+\infty }\int_{-1}^{1}H(\theta )p(\theta \left|x\right.)d\lambda dadb$$

- Absolute Error Loss Function

The absolute error loss function (AEL) penalizes deviations linearly and is less sensitive to large errors than the squared error loss function. Its form is

$$L\left(\hat{H},H\right)=\left|\hat{H}-H\right|$$

In Bayesian inference, the optimal Bayesian estimator ${\hat{H}}_{AEL}$ under the absolute error loss function is the median of the posterior distribution $\pi \left(H\left|x\right.\right)$ of Shannon entropy. Accordingly, the point estimate ${\hat{H}}_{AEL}$ is the unique value η satisfying the following condition:

(45) $${\hat{H}}_{AEL}=\eta$$

where η is the unique value such that the cumulative posterior probability of Shannon entropy ${\hat{H}}_{AEL}$ reaches 0.5, i.e., $\int_{-\infty }^{\eta }p\left(H(\theta )\left|x\right.\right)dH(\theta )=0.5$. Here, x denotes the observed Progressive Type-II censored sample data, and $p\left(H(\theta )\left|x\right.\right)$ is the posterior probability density function of Shannon entropy.

- 0–1 Loss Function

The 0–1 loss function is a type of asymmetric loss function in which the loss is zero only when the estimate equals the true value, and one otherwise. Its form is

$$L_{0-1}\left(H,\overset{\wedge }{H}\right)=\left\{\begin{array}{c} 0,\;if\;\overset{\wedge }{H}=H\\ 1,\;if\;\overset{\wedge }{H}\neq H \end{array}\right.$$

In Bayesian decision theory, the optimal Bayesian point estimator under the 0–1 loss function is defined as the mode of the posterior probability density function of Shannon entropy. Its essence lies in identifying the value at which the posterior probability density function $p\left(H(\theta )\left|x\right.\right)$ attains its maximum over all possible entropy values.

(46) $${\hat{H}}_{0-1L}=\underset{H(\theta )}{\arg\max}\left(p\left(H(\theta )\left|x\right.\right)\right)$$

As noted above, due to the complexity of the TWD likelihood function, the commonly used prior distributions, such as the gamma prior and uniform prior, generally do not form conjugate relationships with the likelihood function, causing the analytical form of the joint posterior distribution $p\left(\theta |x\right)$ to be highly complex; direct integration to obtain marginal posterior distributions or posterior expectations is therefore infeasible. This paper introduces the Markov Chain Monte Carlo (MCMC) method for numerical computation. The MCMC method generates accurate samples from the target posterior distribution to achieve precise and robust Bayesian inference. Based on this algorithm, this study successfully implements reliable Bayesian estimation of the TWD parameters and performs precise measurement and uncertainty quantification of Shannon entropy.

### 4.2. MCMC Algorithm Implementation

Since the likelihood function of the Transmuted Weibull Distribution is analytically intractable, and the commonly adopted prior distributions, such as the Gamma and Uniform priors, are generally not conjugate to the likelihood function, the resulting joint posterior distribution p(θ∣x) does not admit a closed-form expression. Consequently, the marginal posterior distributions and posterior expectations cannot be obtained analytically. To overcome this difficulty, this paper employs the Markov Chain Monte Carlo (MCMC) method for posterior computation. The MCMC method generates samples from the target posterior distribution by constructing a Markov chain whose stationary distribution coincides with the posterior distribution. After a sufficiently large number of iterations, the generated samples can be regarded as approximate draws from the target posterior distribution and can therefore be used for Bayesian inference. Among the various MCMC algorithms, Gibbs sampling is one of the most widely used approaches, in which each parameter is successively sampled from its full conditional posterior distribution given the current values of all remaining parameters. However, for the Transmuted Weibull Distribution, the full conditional posterior distributions of the model parameters do not belong to any standard probability distribution, preventing the direct implementation of a pure Gibbs sampler. Therefore, this paper adopts a hybrid Gibbs sampling algorithm by incorporating Metropolis–Hastings (M–H) updates into the Gibbs sampling framework for those parameters whose full conditional posterior distributions do not have standard closed-form expressions. This hybrid strategy combines the computational efficiency of Gibbs sampling with the flexibility of the Metropolis–Hastings algorithm, thereby enabling efficient posterior sampling and reliable Bayesian inference for both the model parameters and the Shannon entropy.

From the joint posterior distribution (42), the full conditional distributions of the three parameters are, respectively, as follows:

(47) $$\begin{array}{l} p\left(a|b,\lambda ,x\right)\propto a^{m+a_{0}-1}e^{-b_{0}a}\cdot b^{-ma}\cdot {\left(\prod_{i=1}^{m}x_{i}\right)}^{a-1}\cdot \\ \prod_{i=1}^{m}\left[e^{-{\left(\frac{x_{i}}{b}\right)}^{a}}\left(1-\lambda +2\lambda e^{-{\left(\frac{x_{i}}{b}\right)}^{a}}\right){\left(1+\lambda e^{-{\left(\frac{x_{i}}{b}\right)}^{a}}\right)}^{R_{i}}\right] \end{array}$$

(48) $$\begin{array}{l} p\left(b|a,\lambda ,x\right)\propto b^{c_{0}-1-ma}e^{-d_{0}b}\cdot \\ \prod_{i=1}^{m}\left[e^{-{\left(\frac{x_{i}}{b}\right)}^{a}}\left(1-\lambda +2\lambda e^{-{\left(\frac{x_{i}}{b}\right)}^{a}}\right){\left(1+\lambda e^{-{\left(\frac{x_{i}}{b}\right)}^{a}}\right)}^{R_{i}}\right] \end{array}$$

(49) $$p\left(\lambda |a,b,x\right)\propto \prod_{i=1}^{m}\left[\left(1-\lambda +2\lambda e^{-{\left(\frac{x_{i}}{b}\right)}^{a}}\right){\left(1-\lambda +\lambda e^{-{\left(\frac{x_{i}}{b}\right)}^{a}}\right)}^{R_{i}}\right]$$

From Equations (47)–(49), it can be seen that the full conditional distributions of θ are all non-standard in form; the hybrid Gibbs sampling is therefore employed to obtain parameter samples. For parameters a and b, which must take positive values, this paper constructs proposal distributions in the log space by letting $\tilde{a}=\ln a,\tilde{b}=\ln b$, thereby avoiding the issue of proposals falling in the negative domain and eliminating the need to truncate the proposal distribution. For parameter λ, normal proposal distributions are applied directly in the original space, with the constraint that the parameter satisfies the boundary conditions enforced during acceptance probability computation. The specific algorithmic steps are as follows.

Step 1: Set the initial parameter values $\theta^{\left(0\right)}=\left(a^{\left(0\right)},b^{\left(0\right)},\lambda^{\left(0\right)}\right)$, typically taking the maximum likelihood estimates as the starting point. Set the total number of iterations M and the burn-in length $M_{0}$.

Step 2: Let the parameter values at the i-th iteration be $\theta^{\left(i\right)}=\left(a^{\left(i\right)},b^{\left(i\right)},\lambda^{\left(i\right)}\right)$; the procedure for the i+1 iteration is as follows:

(2.1) Sample $a^{\left(i+1\right)}$ using the M-H algorithm. In the log space, generate a candidate value $N\left(\ln a^{\left(i\right)},\sigma_{a}^{2}\right)$ using ${\tilde{a}}^{*}∼N\left(\ln a^{\left(i\right)},\sigma_{a}^{2}\right)$ as the proposal distribution, and let $a^{*}=e^{{\tilde{a}}^{*}}$, where $\sigma_{a}^{2}$ is the $\left(1,1\right)$ element of the inverse observed Fisher information matrix. Compute the acceptance probability:

(50) $$p_{1}=\min\left\{1,\;\frac{p\left(a^{*}|b^{\left(i\right)},\lambda^{\left(i\right)},x\right)}{p\left(a^{\left(i\right)}|b^{\left(i\right)},\lambda^{\left(i\right)},x\right)}\cdot \frac{a^{*}}{a^{\left(i\right)}}\right\}$$

where $\frac{a^{*}}{a^{\left(i\right)}}$ is the Jacobian correction term introduced by the log-space transformation. Draw a random number $u_{1}$ from the uniform distribution $U\left(0,1\right)$ and let

(51) $$a^{\left(i+1\right)}=\left\{\begin{array}{ll} a^{*}, & u_{1}\leq p_{1}\\ a^{\left(i\right)}, & u_{1}>p_{1} \end{array}\right.$$

(2.2) Sample $b^{\left(i+1\right)}$ u using the M-H algorithm. In the log space, generate a candidate value ${\tilde{b}}^{*}∼N\left(\ln b^{\left(i\right)},\sigma_{b}^{2}\right)$ using $N\left(\ln b^{\left(i\right)},\sigma_{b}^{2}\right)$ as the proposal distribution, and let $b^{*}=e^{{\tilde{b}}^{*}}$ where $\sigma_{b}^{2}$ is the (2,2) element of the inverse observed Fisher information matrix. Compute the acceptance probability:

(52) $$p_{2}=\min\left\{1,\;\frac{p\left(a^{\left(i+1\right)},b^{*}|\lambda^{\left(i\right)},x\right)}{p\left(a^{\left(i+1\right)},b^{\left(i\right)}|\lambda^{\left(i\right)},x\right)}\cdot \frac{b^{*}}{b^{\left(i\right)}}\right\}$$

Draw a random number $u_{2}$ from the uniform distribution $U\left(0,1\right)$, and let

(53) $$b^{\left(i+1\right)}=\left\{\begin{array}{ll} b^{*}, & u_{2}\leq p_{2}\\ b^{\left(i\right)}, & u_{2}>p_{2} \end{array}\right.$$

(2.3) Sample $\lambda^{\left(i+1\right)}$ using the M-H algorithm. The proposal distribution for λ is chosen as the normal distribution $N\left(\lambda^{\left(i\right)},\sigma_{\lambda }^{2}\right)$, where $\sigma_{\lambda }^{2}$ is the $\left(3,3\right)$ element of the inverse observed Fisher information matrix.

(i) Draw a candidate value $\lambda^{*}$ from $N\left(\lambda^{\left(i\right)},\sigma_{\lambda }^{2}\right)$; if $\lambda^{*}$, if $\lambda^{*}\notin \left(-1,1\right)$, redraw. Compute the acceptance probability:

(54) $$p_{3}=\min\left\{1,\;\frac{p\left(a^{\left(i+1\right)},b^{\left(i+1\right)},\lambda^{*}|x\right)}{p\left(a^{\left(i+1\right)},b^{\left(i+1\right)},\lambda^{\left(i\right)}|x\right)}\right\}$$

(ii) Draw a random number $u_{3}$ from the uniform distribution $U\left(0,1\right)$, and let

(55) $$\lambda^{\left(i+1\right)}=\left\{\begin{array}{ll} \lambda^{*}, & u_{3}\leq p_{3}\\ \lambda^{\left(i\right)}, & u_{3}>p_{3} \end{array}\right.$$

Step 3: Substitute the current parameter values $\theta^{\left(i+1\right)}=\left(a^{\left(i+1\right)},b^{\left(i+1\right)},\lambda^{\left(i+1\right)}\right)$ into the Shannon entropy expression (11) to compute the corresponding entropy value $H^{\left(i+1\right)}$. Let i=i+1, return to Step 2, and repeat until M iterations are completed.

Step 4: Discard the first $M_{0}$ burn-in samples and retain the remaining $M-M_{0}$ effective samples for subsequent Bayesian estimation and interval estimation.

### 4.3. Bayesian Estimation of Shannon Entropy

After the MCMC chain converges and the burn-in samples are discarded, the remaining $M-M_{0}$ effective parameter samples $\left(a^{\left(t\right)},b^{\left(t\right)},\lambda^{\left(t\right)}\right)$, $t=M_{0}+1,\ldots ,M$, are substituted one by one into Equation (11) to obtain the corresponding Shannon entropy samples $H^{\left(t\right)}=H\left(a^{\left(t\right)},b^{\left(t\right)},\lambda^{\left(t\right)}\right)$ Under the squared error loss function, the Bayesian estimate of Shannon entropy is the posterior mean, obtained by computing the arithmetic mean of the effective samples:

(56) $${\hat{H}}_{\mathrm{SEL}}=\frac{1}{M-M_{0}}\sum_{t=M_{0}+1}^{M}H^{\left(t\right)}$$

Under the absolute error loss function, the Bayesian estimate of Shannon entropy is the posterior median, obtained as the sample median of the effective entropy samples:

(57) $${\hat{H}}_{\mathrm{AEL}}=\mathrm{median}\left\{H^{\left(M_{0}+1\right)},\ldots ,H^{\left(M\right)}\right\}$$

Under the 0–1 loss function, the Bayesian estimate of Shannon entropy is the posterior mode. Kernel density estimation is applied to the effective entropy samples to construct a smooth approximation $\hat{f}\left(h\right)$ of the posterior density, and the peak location of the kernel density estimate curve is taken as an approximation of the posterior mode:

(58) $${\hat{H}}_{0–1}=\arg\max_{h}\hat{f}(h)$$

### 4.4. Highest Posterior Density Credible Interval for Shannon Entropy

In Bayesian inference, the highest posterior density (HPD) credible interval is widely used because it provides the shortest interval for a given posterior probability and is particularly suitable for asymmetric posterior distributions. Based on the posterior samples generated by the hybrid Gibbs sampling algorithm described in Section 4.2, the HPD credible interval is constructed according to the following steps.

(1) Obtain Shannon entropy samples. From Steps 3 and 4 of Section 4.2, $N=M-M_{0}$ effective posterior samples of Shannon entropy ${\left\{H^{\left(t\right)}\right\}}_{M_{0}+1}^{M}$ have already been obtained.

(2) Sort in ascending order. Arrange the above samples from smallest to largest, denoted as

(59) $$H_{\left(1\right)}\leq H_{\left(2\right)}\leq \cdots \leq H_{\left(N\right)}$$

(3) Construct the HPD credible interval. For a given significance level γ, compute the number of samples that the interval should contain:

(60) $$k=\left\lfloor N\times (1-\gamma )\right\rfloor$$

Iterate over all possible starting points j=1,2,…,N−k, and compute the length of each candidate interval:

(61) $$L_{j}=H_{\left(j+k\right)}-H_{\left(j\right)}$$

Select the index $j^{*}$ that minimizes $L_{j}$:

(62) $$j^{*}=\arg\min_{1\leq j\leq N-k}L_{j}$$

Then, the $100\left(1-\gamma \right)\%HPD$ HPD credible interval for Shannon entropy is

(63) $$\left(H_{\left(j^{*}\right)},H_{\left(j^{*}+k\right)}\right)$$

## 5. Monte Carlo Simulation Study

Monte Carlo simulation studies are conducted to evaluate the finite-sample performance of the proposed inferential framework. Under various combinations of sample sizes and Progressive Type-II censoring schemes, the statistical properties of the maximum likelihood and Bayesian estimators are systematically compared. Estimation performance is assessed in terms of bias, mean squared error (MSE), and interval estimation accuracy, while the effects of different loss functions on the Bayesian entropy estimators are also investigated. The simulation results provide comprehensive numerical evidence for assessing the accuracy, efficiency, and robustness of the proposed inferential procedures under different censoring scenarios.

### 5.1. Numerical Computation Methods

In Monte Carlo simulation studies, Progressive Type-II censored samples need to be generated. Specifically, the steps for generating Progressive Type-II censored data from $X∼TW\left(a_{real},b_{real},\lambda_{real}\right)$ are as follows, where $a_{real},b_{real},\lambda_{real}$ are the true parameter values set for the simulation:

(1) Generate m random numbers from the uniform distribution $U\left(0,1\right)$, denoted as $w_{1},w_{2},\ldots ,w_{m}$.
(2) First let $v_{i}=w_{i}^{{\left(i+R_{m}+R_{m-1}+\ldots +R_{m-i+1}\right)}^{-1}}$, then let $u_{i}=1-v_{m}v_{m-1}\ldots v_{m-i+1}$, where i=1,2,…,m.
(3) Let $x_{i}=F^{-1}\left(u_{i};a_{real},b_{real},\lambda_{real}\right)$; then $\left(x_{1},x_{2},\ldots ,x_{m}\right)$ is the Progressive Type-II censored data from $TW\left(a_{real},b_{real},\lambda_{real}\right)$ with censoring scheme $\left(R_{1},R_{2},\ldots ,R_{m}\right)$, where F(x) is the cumulative distribution function of the Transmuted Weibull distribution.

### 5.2. Simulation Design

Configure the MCMC algorithm parameters (see Table 1).

Configure the PC-II solution (see Table 2).

We set a=2,b=1,λ=0.5, along with hyperparameters under the gamma prior $a_{0}=1,b_{0}=1,c_{0}=1,d_{0}=1$. The true value of Shannon entropy is 0.4759558028. Table 3 reports the deviations and mean squared errors of the maximum likelihood and Bayesian estimates of the Shannon entropy under different censoring schemes.

(1) MLE exhibits significant negative bias in all settings, whereas the absolute bias of the three Bayesian estimators is markedly smaller, indicating higher estimation precision.
(2) In terms of MSE, the Bayesian estimators outperform MLE across all combinations of sample size and censoring scheme, and this advantage becomes particularly pronounced when the censoring proportion is high.
(3) As the sample size n increases or the number of effective observations m increases, the bias and MSE of all methods show a declining trend, with the Bayesian estimators converging more rapidly.

Based on the table above, the following conclusions can be drawn:

(1) All three interval estimation methods achieve coverage probabilities close to or above the nominal 95% level in most cases, confirming the reliability of the proposed procedures under progressive Type-II censoring.
(2) The HPD credible intervals consistently exhibit competitive coverage probabilities while maintaining narrower interval widths compared to ACI in the majority of configurations, suggesting that the Bayesian approach provides more precise interval estimation.
(3) The Bootstrap intervals tend to yield coverage probabilities that are occasionally higher than nominal, particularly in small-sample settings (e.g., n=30,m=8), which may reflect the conservative nature of the percentile bootstrap under high censoring rates.
(4) As the effective sample size m increases or the total sample size n grows, the interval widths generally narrow across all three methods, which is consistent with the expected asymptotic behavior.

## 6. Empirical Analysis

Several criteria exist in the literature for evaluating the performance of statistical inference on entropy. This paper adopts bias, mean square error, and confidence interval coverage rate as evaluation metrics to examine the effectiveness of the proposed estimation methods. Given a dataset, the method that simultaneously achieves the minimum bias and mean square error while yielding a coverage rate closest to the nominal confidence level is regarded as the optimal estimation method. To validate the performance of the Bayesian estimation methods proposed in this paper, maximum likelihood estimation is selected as the comparative benchmark. The dataset used is a random sample comprising the remission times (in months) of 128 bladder cancer patients (Lee and Wang, 2003 [31]).

**Figure 3:**
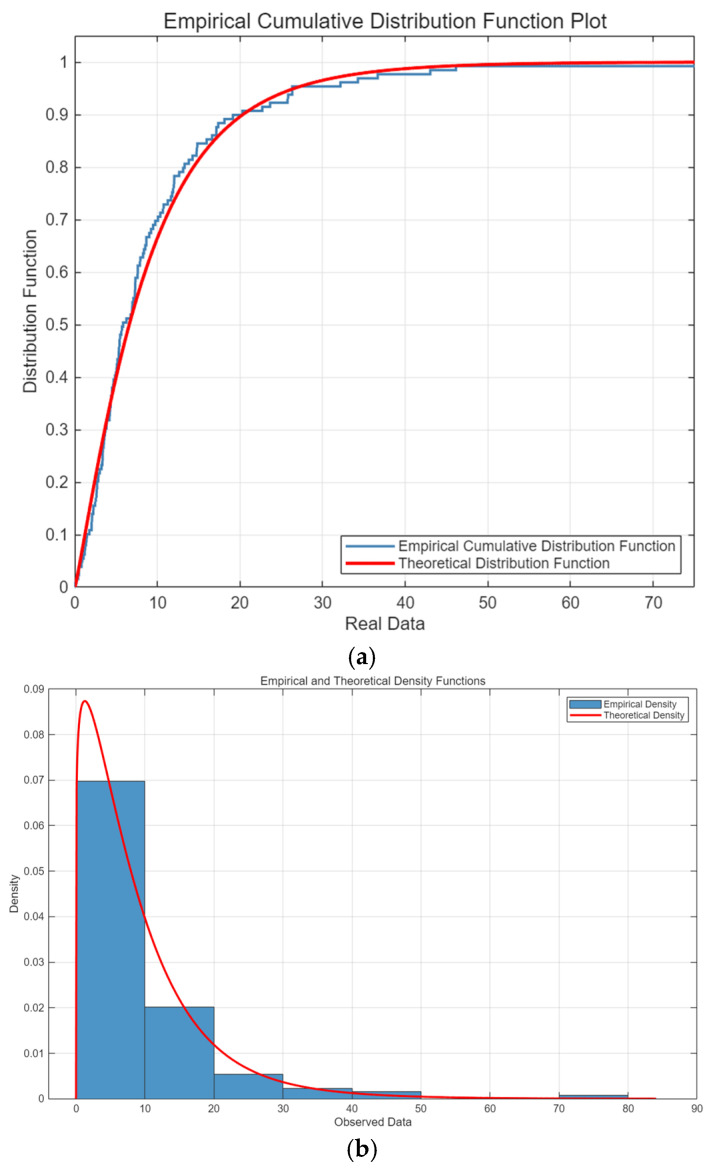

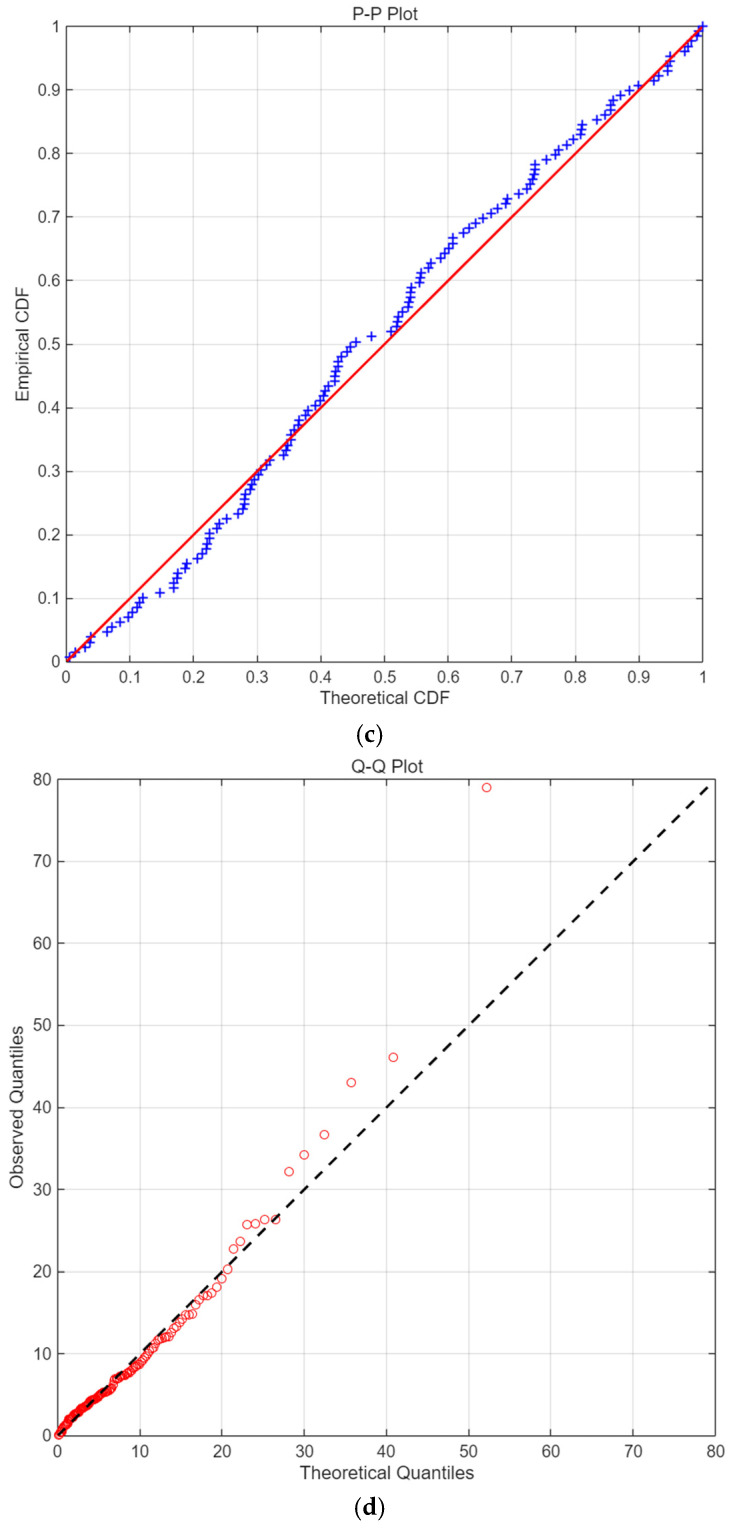
Goodness-of-fit plot for the TWD model using data on response duration in bladder cancer patients: (**a**) empirical cumulative distribution function (ECDF) with the fitted TWD cumulative distribution function; (**b**) empirical histogram with the fitted TWD probability density function; (**c**) P–P plot; and (**d**) Q–Q plot.

To further verify the effectiveness of the Transmuted Weibull Distribution in fitting the remission time data of bladder cancer patients, a more intuitive visualization approach is adopted in this paper, as shown in Figure 3.

To further validate and illustrate the performance of the statistical inference method proposed in this paper in practical applications, we have devised three different censoring schemes, A, B and C, as shown in Table 4, Table 5, Table 6, Table 7 and Table 8. In addition, the confidence intervals for the MLE and Bayesian estimates in the cancer dataset are reported in Table 9.

**Figure 4:**
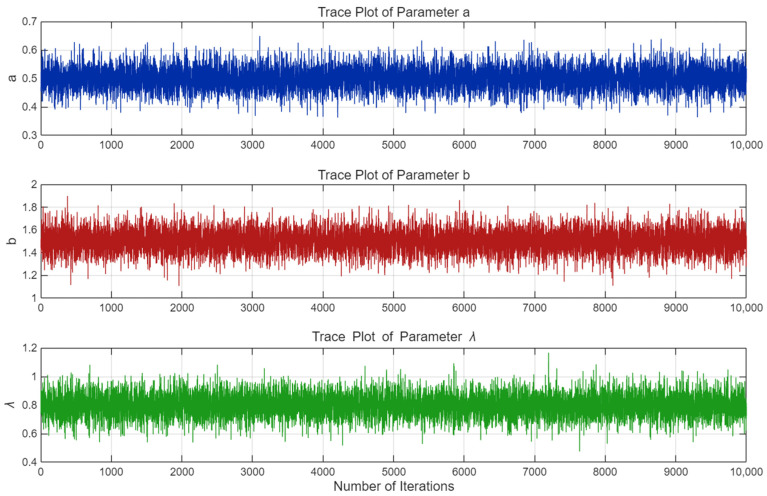
Plot of parameter trajectories during the MCMC sampling process.

**Figure 5:**
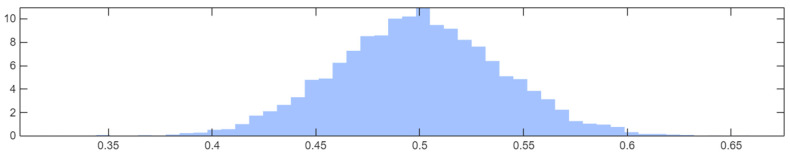
Posterior Distribution Histograms of Parameters Based on MCMC Samples.

To verify whether the Markov chains generated during the MCMC sampling process have reached an ideal state of convergence, this paper uses samples generated from a bladder cancer patient dataset under different censoring schemes to perform a convergence diagnosis. Under the conditions of a given gamma prior and a uniform prior, Figure 4 and Figure 5 illustrates the parameter trajectories of these samples during the sampling process.

## 7. Conclusions

This paper systematically investigates statistical inference for the Shannon entropy of the Transmuted Weibull Distribution under Progressive Type-II censored samples and develops a comprehensive inferential framework integrating both frequentist and Bayesian approaches. The proposed framework is further evaluated through Monte Carlo simulation studies and illustrated using a real medical dataset.

From a frequentist perspective, the log-likelihood function of the Transmuted Weibull Distribution is established based on Progressive Type-II censored samples, and the maximum likelihood estimators of the model parameters and Shannon entropy are obtained using the Newton–Raphson algorithm. The asymptotic variance of the Shannon entropy estimator is derived using the Delta method, and both asymptotic confidence intervals and Bootstrap confidence intervals are constructed for interval estimation. The simulation results show that the maximum likelihood estimator exhibits systematic negative bias across all parameter settings and censoring schemes. This bias becomes more pronounced for small sample sizes and high censoring proportions, leading to a deterioration in estimation performance under complex censoring scenarios.

Within the Bayesian framework, independent Gamma priors are assigned to the shape and scale parameters, while a Uniform prior is assigned to the transmutation parameter. Posterior inference is carried out via a hybrid Gibbs sampling algorithm within the MCMC framework. Bayesian point estimators of Shannon entropy are obtained under the squared error, absolute error, and 0–1 loss functions, corresponding to the posterior mean, posterior median, and posterior mode, respectively. Monte Carlo simulation results show that all three Bayesian estimators consistently outperform the MLE in terms of both bias and MSE. Moreover, as the sample size increases or the degree of censoring decreases, the estimators exhibit faster convergence and improved accuracy. For interval estimation, HPD credible intervals are constructed for the Shannon entropy of the TWD and compared with ACI and Bootstrap confidence intervals. The results indicate that HPD credible intervals achieve the most stable coverage probability and the shortest average interval length across different censoring schemes and sample size settings, demonstrating superior overall performance.

An empirical analysis based on the remission times of 128 bladder cancer patients further supports the proposed inferential framework. Under three censoring schemes, the Bayesian estimators yield lower bias and MSE compared with the MLE. In addition, the HPD credible intervals achieve higher coverage probabilities than both the ACI and Bootstrap intervals. These findings are consistent with the simulation results and confirm the practical applicability of the proposed methods.

The present study develops a comprehensive inferential framework for entropy estimation under complex censored samples and provides a Bayesian-based methodology for uncertainty quantification in highly reliable systems and medical survival data analysis. Future work may extend the proposed approach to other generalized entropy measures, such as Rényi entropy and Tsallis entropy, or to more complex data structures, including competing risks models and adaptive censoring schemes, thereby broadening its applicability to a wider range of reliability and survival analysis problems.

## Figures and Tables

**Table 1:** Algorithmic configurations and parameter settings for the MCMC procedure.

Item	Setting
Monte Carlo replications	1000
Total MCMC iterations	10,000
Burn-in period	5000
a	$lna^{*}∼N(lna^{\left(i\right)},\sigma_{a}^{2})$
b	$lnb^{*}∼N(lnb^{\left(i\right)},\sigma_{b}^{2})$
λ	$\lambda^{*}$∼N(λ(i),σλ2)
$\sigma_{a},\sigma_{b},\sigma_{\lambda }$	Square roots of the (1,1), (2,2), (3,3) diagonal elements of $I^{-1}({\hat{\theta }}_{MLE})$
Observed acceptance rates	38.69% (a), 51.24% (b), 52.66% (λ)

**Table 2:** Three different censoring schemes.

Scheme	
A	$R_{1}=R_{2}=\cdots =R_{m-1}=0,R_{m}=n-m-\sum_{i=1}^{m-1}R_{i}$
B	$R_{1}=R_{2}=R_{3}=\cdots =R_{m-2}=0,R_{m-1}=R_{m}=\frac{n-m}{2}$
C	$R_{1}=R_{2}=\cdots =R_{m-2}=0,R_{m}=0,R_{m-1}=n-m-\sum_{i=1}^{m-1}R_{i}$

**Table 3:** Deviations and mean squared errors of the maximum likelihood and Bayesian estimates of entropy under different censoring schemes.

			MLE		SEL		AEL		0–1 L	
n	m	CS	Bias	MSE	Bias	MSE	Bias	MSE	Bias	MSE
30		A	−0.4183	0.4517	0.0656	0.1648	−0.0034	0.2211	0.0728	0.1620
	8	B	−0.4445	0.4535	0.0524	0.1630	0.0929	0.1578	0.1021	0.1205
		C	−0.4110	0.3925	0.0893	0.1590	0.0275	0.1525	−0.0478	0.1384
		A	−0.2298	0.1285	0.0070	0.0619	−0.0158	0.0895	−0.0213	0.0585
	16	B	−0.2483	0.1362	0.0174	0.0685	−0.0222	0.0614	−0.0168	0.0676
		C	−0.2065	0.1249	0.0116	0.0703	−0.0270	0.0620	−0.0012	0.0620
		A	−0.1564	0.0642	0.0039	0.0305	−0.0353	0.0274	−0.0330	0.0243
	24	B	−0.1474	0.0584	0.0213	0.0311	−0.0032	0.0392	−0.0215	0.0289
		C	−0.1437	0.0632	−0.0209	0.0276	−0.0192	0.0209	−0.0134	0.0204
40		A	−0.4560	0.5241	0.1118	0.1890	−0.0489	0.1992	−0.0263	0.1635
	8	B	−0.4511	0.4931	0.0951	0.2069	0.0738	0.1760	0.0525	0.2030
		C	−0.4096	0.3910	0.0818	0.1698	−0.0173	0.1415	0.0358	0.1745
		A	−0.2449	0.1640	0.0250	0.0629	0.0202	0.0799	−0.0497	0.0796
	16	B	−0.2683	0.1705	−0.0005	0.0851	0.0009	0.0553	0.0044	0.0759
		C	−0.3026	0.2106	0.0072	0.0765	0.0252	0.0795	−0.0314	0.0607
		A	−0.1963	0.0832	0.0242	0.0398	−0.0411	0.0401	0.0158	0.0433
	24	B	−0.2032	0.0873	−0.0181	0.0432	−0.0070	0.0347	−0.0327	0.0438
		C	−0.1786	0.0758	0.0175	0.0347	−0.0127	0.0328	−0.0250	0.0336
50		A	−0.4632	0.6040	0.1242	0.1669	−0.0617	0.1708	0.0812	0.1627
	8	B	−0.5016	0.6041	0.0898	0.1683	0.0574	0.1729	−0.0022	0.1711
		C	−0.4560	0.4630	0.0868	0.1426	0.0375	0.1438	−0.0291	0.1463
		A	−0.2529	0.1820	0.0458	0.0948	−0.0023	0.0920	0.0384	0.0622
	16	B	−0.2613	0.1828	0.0060	0.1025	−0.0134	0.0887	0.0079	0.0569
		C	−0.2814	0.1883	0.0166	0.0754	−0.0094	0.0683	0.0092	0.0807
		A	−0.1990	0.0900	0.0056	0.0481	0.0092	0.0569	−0.0384	0.0475
	24	B	−0.1943	0.0911	0.0075	0.044	0.0253	0.0287	−0.0235	0.0359
		C	−0.1837	0.0887	0.0041	0.039	−0.0430	0.0475	−0.0064	0.0383

**Table 4:** Confidence intervals and coverage of estimates of maximum likelihood and Bayesian entropy under different censoring schemes.

			ACI		Bootstrap		HPD	
n	m	CS	CI	CP(%)	CI	CP(%)	CI	CP(%)
30		A	[−0.2109, 1.7400]	97.00	[0.0424, 1.8771]	98.98	[0.4599, 1.3348]	97.00
	8	B	[−0.2913, 1.6485]	98.00	[0.3390, 2.0475]	98.00	[0.4040, 1.3430]	96.00
		C	[−0.2146, 1.5920]	99.00	[0.2235, 2.1626]	100.00	[0.7137, 1.3615]	98.00
		A	[0.0278, 1.0935]	99.00	[0.1146, 1.8783]	99.00	[0.4413, 1.6128]	98.00
	16	B	[0.0444, 1.1112]	96.00	[0.0173, 1.0785]	97.00	[0.2594, 1.9446]	97.00
		C	[0.0311, 1.0568]	98.00	[0.0219, 1.9459]	100.00	[0.4531, 1.8150]	97.00
		A	[0.2025, 0.8794]	93.00	[0.0274, 1.5616]	99.00	[0.4265, 1.3992]	98.00
	24	B	[0.1602, 0.8337]	94.00	[0.0176, 0.3548]	93.00	[0.2733, 1.8559]	96.00
		C	[0.1830, 0.8524]	97.00	[0.0123, 0.8045]	95.00	[0.5340, 1.4413]	97.00
40		A	[−0.3432, 1.7926]	100.00	[0.4586, 1.6498]	94.00	[0.4228, 1.9450]	96.00
	8	B	[−0.3451, 1.7391]	100.00	[0.0484, 1.3747]	93.00	[0.6121, 1.6743]	97.00
		C	[−0.2726, 1.6113]	99.00	[0.1166, 1.2711]	94.00	[0.0048, 1.3670]	98.00
		A	[−0.0718, 1.1460]	98.00	[0.1499, 1.9407]	95.00	[0.4413, 1.2631]	98.00
	16	B	[−0.0298, 1.1767]	97.00	[0.0200, 1.7045]	94.00	[0.4557, 1.6861]	94.00
		C	[−0.0157, 1.1585]	98.00	[0.0313, 1.7995]	100.00	[0.3220, 1.8140]	99.00
		A	[0.1110, 0.9170]	94.00	[0.0503, 1.2288]	99.00	[0.4413, 1.2451]	97.00
	24	B	[0.1195, 0.9214]	97.00	[0.0426, 0.8734]	95.00	[0.2254, 1.1897]	96.00
		C	[0.1389, 0.9250]	97.00	[0.0164, 1.6691]	100.00	[0.3931, 1.5919]	96.00
50		A	[−0.3509, 1.8387]	98.00	[0.4271, 1.7110]	94.00	[0.4413, 1.1035]	98.00
	8	B	[−0.3514, 1.8197]	98.00	[0.0427, 1.7174]	99.00	[0.6827, 1.2784]	97.00
		C	[−0.2496, 1.6466]	97.00	[0.1711, 1.5871]	93.00	[0.1791, 1.1845]	96.00
		A	[−0.0857, 1.2388]	98.00	[0.1483, 1.7413]	98.00	[0.4413, 1.6272]	98.00
	16	B	[−0.0684, 1.2568]	98.00	[0.0219, 1.7254]	99.00	[0.5613, 1.9085]	96.00
		C	[−0.0214, 1.2367]	97.00	[0.0586, 1.7221]	100.00	[0.1346, 1.0417]	98.00
		A	[0.0761, 0.9852]	96.00	[0.0646, 1.8536]	99.00	[0.4413, 1.2808]	98.00
	24	B	[0.0871, 0.9961]	96.00	[0.0161, 1.3029]	97.00	[0.4092, 1.0654]	96.00
		C	[0.0882, 0.9541]	94.00	[0.0282, 1.1645]	95.00	[0.3340, 1.6383]	97.00

**Table 5:** MLE and entropy estimates of parameters for the cancer dataset under different censoring schemes.

CS	a	b	λ	Shannon Entropy
A	1.1254	6.0841	−0.6105	2.9447
B	1.1371	6.1161	−0.5963	3.4150
C	1.1484	6.1463	−0.5826	3.2363

**Table 6:** SEL and entropy estimates for parameters in the cancer dataset under different censoring schemes.

CS	a	b	λ	Shannon Entropy
A	1.2752	8.8294	0.0482	2.9730
B	0.9372	10.9309	−0.0945	3.3712
C	0.9737	10.2741	−0.0361	3.2320

**Table 7:** AEL and entropy estimates for parameters in the cancer dataset under different censoring schemes.

CS	a	b	λ	Shannon Entropy
A	1.2908	8.4245	0.0583	2.9689
B	0.9569	10.3154	−0.0063	3.3629
C	0.9899	10.4762	0.1876	3.2271

**Table 8:** 0–1 L and entropy estimates for parameters in the cancer dataset under different censoring schemes.

CS	a	b	λ	Shannon Entropy
A	1.2761	6.6565	0.5300	2.9490
B	1.0746	6.0255	−0.9500	3.3425
C	1.0753	5.4330	−0.9700	3.2125

**Table 9:** Confidence intervals for MLE and Bayesian estimates in the cancer dataset.

CS	ACI	Length	Bootstrap	Length	HPD	Length
A	[1.7419, 2.3946]	0.6527	[1.9944, 2.3051]	0.3107	[1.8806, 2.4697]	0.5891
B	[1.7536, 2.2874]	0.5338	[1.7449, 2.2954]	0.5505	[1.9347, 2.3333]	0.3986
C	[2.0115, 2.5401]	0.5286	[1.8265, 2.8748]	1.0483	[1.7772, 2.5255]	0.7483

## Data Availability

Data are contained within the article.
